# Active Surveillance for Highly Pathogenic Avian Influenza Viruses in Wintering Waterbirds in Northeast Italy, 2020–2021

**DOI:** 10.3390/microorganisms9112188

**Published:** 2021-10-20

**Authors:** Federica Gobbo, Diletta Fornasiero, Maria Alessandra De Marco, Bianca Zecchin, Paolo Mulatti, Mauro Delogu, Calogero Terregino

**Affiliations:** 1 Comparative Biomedical Sciences Division, Istituto Zooprofilattico Sperimentale delle Venezie, 35020 Legnaro, PD, Italy; fgobbo@izsvenezie.it (F.G.); bzecchin@izsvenezie.it (B.Z.); 2 Veterinary Epidemiology Unit, Laboratory of Epidemiological Surveillance and Veterinary Legislation, Istituto Zooprofilattico Sperimentale delle Venezie, 35020 Legnaro, PD, Italy; dfornasiero@izsvenezie.it (D.F.); pmulatti@izsvenezie.it (P.M.); 3 Institute for Enviromental Protection and Research (ISPRA), 40064 Ozzano dell’Emilia, BO, Italy; mariaalessandra.demarco@isprambiente.it; 4 Wildlife and Exotic Animal Service, Department of Veterinary Medical Sciences, University of Bologna, 40064 Ozzano dell’Emilia, BO, Italy; mauro.delogu@unibo.it

**Keywords:** avian influenza virus, HPAI H5 subtypes clade 2.3.4.4b, migratory aquatic bird, active surveillance

## Abstract

The increasing involvement of wild waterfowl in H5 Highly Pathogenic Avian Influenza Virus (HPAIV) circulation continues to pose a threat to animal and public health worldwide. In winter 2020–2021, two field surveillance activities were carried out on a weekly basis, through virological and serological analyses, in 823 hunted and 521 trapped migratory aquatic birds in northeast Italy. Sixty Eurasian teals were recaptured several times, which allowed us to follow the progression of the HPAI H5 infection in naturally infected wild waterfowl. Oropharyngeal, cloacal, and feather swabs (OS, CS and FS) were collected from each duck and tested by real time rRT-PCR Type A influenza. The identified viruses were characterized and pathotyped by sequencing. Several viruses belonging to three different HPAI H5 subtypes were detected: H5N8, H5N5, and H5N1. High prevalence of infection with HPAI H5 clade 2.3.4.4b during November–December 2020 (up to 27.1%) was observed in captured Eurasian teals, while infection rates in hunted dabbling ducks, mainly Eurasian wigeons, showed the highest prevalence of infection in November 2020 (8.9%) and January 2021 (10.2%). All HPAI positive birds were also clinically healthy when recaptured weeks apart. The OS and FS showed the highest detection efficiency of HPAIV. Our results highlight that HPAI passive surveillance should be complemented by a targeted active surveillance to more efficiently detect novel HPAI viruses.

## 1. Introduction

It has long been known that wild aquatic birds enable the perpetuation of low-pathogenic avian influenza viruses (LPAIVs) that, in poultry, can occasionally evolve into highly pathogenic (HP) strains. However, in recent years, the increasing involvement of these reservoir hosts in H5 HPAIV circulation and spread has opened a new scenario in which HPAI H5 viruses belonging to clade 2.3.4.4 of the Goose/Guangdong/96 (GS/Gd) lineage pose an even greater threat to poultry and wild birds worldwide. Reassortment events between these HPAI H5 and LPAIVs of wild bird origin have led to generation of different variants that have been periodically spread by wild birds across continents [1,2,3].

In the autumn–winter season of 2020–2021, following the confirmation of several HPAI outbreaks of the H5 subtype among wild and domestic birds in western Russia and Kazakhstan in May–September 2020, all European Union (EU) member states were urged to intensify surveillance activities and to increase biosecurity measures in the poultry sector to avoid new avian influenza outbreaks. Actually, these eastern affected areas are known autumn staging sites for wild water birds heading winter quarters in Europe, and given past experience, it was very likely that a new avian influenza wave might hit northern and eastern Europe in the last fall and winter, and from there spread to southern and western Europe [2]. Prior to the ongoing HPAI H5Nx avian influenza panzootic in Europe, since the winter season of 2005–2006, Italy has been hit by the introduction of other different HPAI viruses [1]. Following the 2017–2018 H5 HPAI epidemic in north Italy, a series of ancillary activities were scheduled to enhance the National Surveillance Plan. In particular, pilot surveillance activities were designed in wetlands, which are considered strategic for the introduction of avian influenza viruses via migratory movements. Although the main objectives of this surveillance effort were to promptly identify the circulating HPAIV in wild birds entering the EU through migration and to provide information on the potential AIV spread with movements of wild birds, these activities have also contributed to provide deeper knowledge about AIV ecology.

In this paper, we report the results obtained in winter 2020–2021 from the active surveillance in wild birds for HPAI in northeast Italy. Two main field activities were organized in wetlands zones considered strategic for early detection of HPAIVs in hunted aquatic birds, with periodic samplings during the hunting season 2020–2021 in wetlands of the provinces of Rovigo and Venice (surveillance activity 1) and an active surveillance in aquatic birds, trapped thanks to the cooperation with ornithologists in two capture stations, located in wetland areas in the province of Venice (surveillance activity 2).

## 2. Materials and Methods

*Background*: Surveillance activities were conducted in accordance with the decree of the Italian Ministry of Health of 14 March 2018 [https://www.gazzettaufficiale.it/eli/gu/2018/04/19/91/sg/pdf; accessed on 24 August 2021], which established a pilot risk-based surveillance program in wild birds, integrating the National Surveillance Plan for Avian Influenza. The efforts were mainly concentrated in northeast Italy, a geographic area previously involved in multiple avian influenza epidemics, which has a unique combination of densely populated poultry areas (DPPAs) and wetlands.

The strategic wetlands available for the active surveillance in wild birds are located along the Eurasian Anatidae flyway, and represent resting and wintering sites for many wild waterfowl migrating south from northern and eastern Europe. In addition, waders migrating south stop to rest and feed in the same wetlands, before undertaking the long flight over the Mediterranean Sea that precedes their over-wintering in Africa [4,5]. The migrant population is largely composed of juveniles, hatched during the previous spring, which are known to be highly susceptible to influenza virus infection. Therefore, during the winter season, thousands of susceptible migratory birds originating from different sites in northern and eastern Europe congregate in these Italian wetlands and intermingle with the resident population, creating an ideal ecological setting for the maintenance and spread of influenza viruses and for the onset of reassortment mechanisms leading to novel viruses.

**Figure 1:**
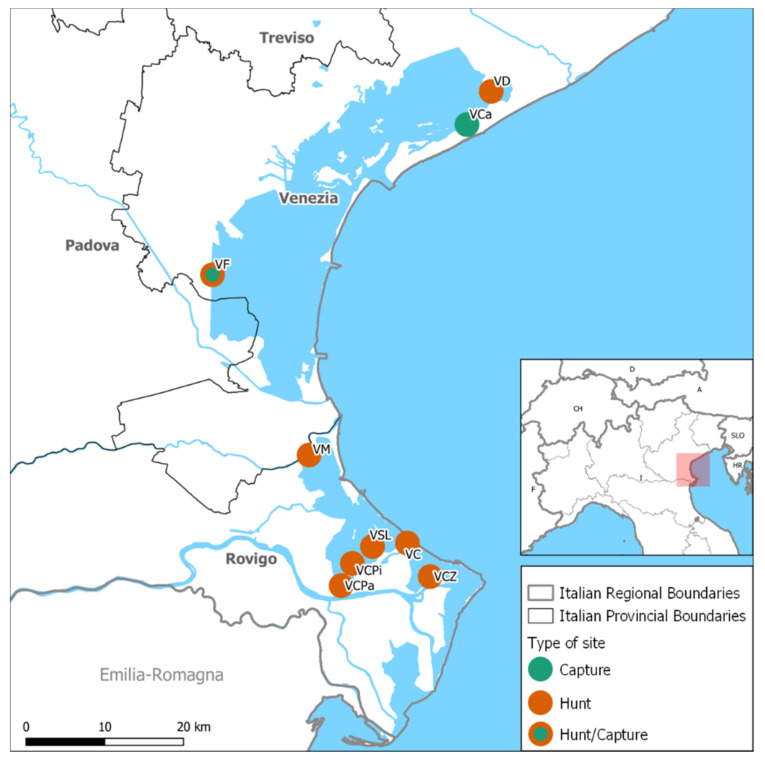
Location of hunting sites (orange color) and capture sites (green color). V, Valle; VCPa, V. Ca Pasta (RO); VCZ, V. Ca Zuliani (RO); VD, V. Drago (VE); VSL, V. San Leonardo (RO); VC, V. Chiusa (RO); VCPi, V. Ca Pisani (RO); VM, V. Morosina (RO); VF, Valle Figheri (VE); VCa, Valle Cavallino.

*Surveillance activity 1 (hunted ducks)*: Sampling activities were conducted from November 2020 to January 2021 in eight wetlands hosting private hunting grounds, located in the Rovigo and Venice provinces (Figure 1), and considered strategic for AI surveillance due to their position along the migratory routes of waterfowl. Access to the hunting grounds was secured by agreements with Regional Authorities and the site manager. During each session, the game bag was subjected to species identification, and sampling via oropharyngeal swabs (OS), cloacal swabs (CS) and feather swabs (FS). Soon after sampling, the specimen were transferred in refrigerated containers to the laboratory for AIV diagnostic investigations. The activities were carried out on a weekly basis between November 2020 and January 2021; due to logistical constraints, it was only possible to visit one single area per week. Field activities were scheduled according to the SARS-CoV-2 emergency, the consequent Italian Government Law disposals, and hunters’ and veterinarians’ availability. The majority of the sampling sites were visited at least twice, with one site (Valle Drago) visited up three times.

From November 2020 to January 2021, 15 samplings were performed in the Veneto wetlands at different sampling periods (Table 1); 823 hunted birds were sampled for a total of 2449 swabs collected and submitted to the laboratory for molecular detection of AIV. The waterfowl species sampled included the Eurasian Teal (ET) *Anas crecca*, Gadwall (Ga) *Mareca strepera*, Northern Pintail (NP) *Anas acuta*, Eurasian Wigeon (EW) *Mareca penelope*, Mallard (Ma) *Anas platyrhynchos* and Northern Shoveler (NS) *Spatula clypeata*. Moreover, post-mortem blood samples were collected via heart clots from 18 hunted EWs for serological analyses.

*Surveillance activity 2 (captured ducks)*: Sampling activities in captured migratory waterfowl were performed from November 2020 to March 2021 at two wetlands located in the province of the Venice: Valle Figheri and Valle Cavallino (Figure 1). The first area is considered a biodiversity hotspot; the presence of both migratory and resident aquatic birds, and the constant support of ornithologists, makes this wetland ideal both for active surveillance of AIV in birds and the environment, and for gaining deeper insight on the ecological aspects of AIV in their natural reservoirs. Fourteen different field samplings were organized in Valle Figheri in the period 12 November 2020–5 February 2021 (Table 2). Valle Cavallino, located in the northern Lagoon of Venice, was also included in the activity for two field sample collections in February 2021, following the evidence of HPAI viruses in some backyard poultry farms.

A total of 521 dabbling ducks were tested and in detail 419 captured birds were seized in the trapping site of Valle Figheri, including the ET, Ga, and Ma species, whereas 102 captured aquatic birds caught in Valle Cavallino included the ET, NP, EW, and Common Shelduck (CSh) *Tadorna tadorna* (Table 2).

Wild waterfowl were captured using Abberton Traps approved by Decrees n° 14 5 February 2018 and n° 33 9 September 2020 of the Veneto Region and according to the approvals of the Italian Institute for Environmental Protection and Research (ISPRA–Istituto Superiore per la Protezione e la Ricerca Ambientale) the Veneto Region Register n° 21,310 18 January 2018, 25,895 23 January 2018 and the Veneto Region Register n° 16,988 14 January 2021. Therefore, birds were handled and sampled by authorized and trained ornithologists and veterinarians, to reduce any potential distress to the birds. Each captured animal was transferred from the traps into wood boxes, ring-tagged, and subjected to weight and morphometric measuring, ageing, and sexing, thus birds were sampled via OS, CS, and FS and then released into the wild after sampling procedures. The collected samples were immediately transported to the laboratory and submitted for AIV diagnostic investigations for a total of 1563 swabs analyzed.

Feather swabs collection, obtained through rubbing the external body surface by the means of a sterile swab, was performed according to Delogu et al. [6].

In Valle Figheri birds were also sampled for blood collection on four different sampling dates (Table 2: VF-4, VF-10, VF-11 and VF-12) to perform serological analyses. Blood sera were collected from 138 Eurasian teals.

*Animal Rights Statements*: No birds were expressly killed or captured for this study. No specific permission was required for sample collection from shot wild birds, killed by local hunters in compliance with the Italian hunting laws. Wild birds were captured alive during authorized ringing activities carried out in the study area.

No supplementary permits or approvals were needed for sampling from wild birds captured alive for ringing activities, because this sampling was conducted as part of the national avian influenza surveillance program. All birds were handled in accordance with “Guidelines to the Use of Wild Birds in Research” [7].

*Molecular detection of AIV*: Briefly, all OS, CS, and FS obtained from surveillance activity 1 and 2 were moved into single tubes containing a sufficient amount of PBS (with antibiotics) to ensure their full immersion, approximately into 1 mL for OS and 2.5 mL for CS and FS, then the swab suspensions were vortexed for 30 s and centrifuged for 2 min at 15,000× *g*. RNA extraction was performed using QIAsymphony DSP Virus/Pathogen Kit (Qiagen) on the QIAsymphony SP instrument and submitted for detection of Influenza A (M-gene) by real-time RT-PCR (rRT-PCR) [8] using Corbett Research Rotor-Gene™ as platform.

All samples that tested positive by rRT-PCR Type A Influenza were promptly tested for the hemagglutinins H5 and H7 by RRT-PCRs [9,10] as well by conventional one-step RT-PCRs [11] in order to pathotype the hemagglutinin cleavage site by Sanger sequencing.

Neuraminidase (NA) typing of avian influenza positive samples was performed by rRT-PCR, using multiple oligonucleotides sets based on the assays developed by Hoffmann et al. [12] and James et al. [13].

All M-gene positive samples and negative for H5 and H7 were also tested for H9 hemagglutinin by rRT-PCR [14].

*Serological analyses*: Blood sera were analyzed for antibodies against type A Influenza using a commercial competitive ELISA (IDVet^®^, Grabels, France) and hemagglutination inhibition (HI) test.

The HI test was carried out with the following HPAI H5 reference antigens, Clade 2.3.4.4b: A/mute swan/Netherlands/01/20, H5N8 and A/wigeon/Italy/16VIR9616-3/16, H5N5. Laboratory methods were performed in compliance with internal procedures [15] and according to the Manual of Diagnostic Tests and Vaccines for Terrestrial Animals OIE [16].

*Virus isolation in SPF embryonated eggs and AIV typing*: In order to verify the vitality of the virus identified in the different types of matrices, selected samples positive for HPAI H5 virus were submitted to virus isolation following the internal laboratory procedures (IZSVe SOP VIR 005) [15], based on those described in the OIE Terrestrial Manual (2021) [16].

Briefly, 9 to 11 day-old SPF (Specific Pathogen Free) embryonated chicken eggs were inoculated into the allantoic cavity with biological samples (five eggs/sample). The eggs were then candled daily to check the embryos’ mortality, while the allantoic fluids harvested from dead embryos were submitted to hemagglutination (HA) and HI testing in order to achieve the AIV subtype.

*Statistical analyses*: Data related to HPAI virus detection were used to assess any information of interest on virus finding in wild birds, keeping separated hunted from captured birds (Activity 1 and 2, respectively). Epidemiological curves were calculated for both activities to explore any potential temporal trend in HPAI occurrence. Furthermore, a series of binomial generalized linear mixed models (GLMMs) were built to estimate any correlation between the swab type used for sampling and the probability of detecting HPAI viruses. The structures of the GLMMs were defined following a series of preliminary univariate linear regressions, separately conducted both on hunted and captured wild birds, to identify the most appropriate random effects to be included. Specifically, sampling area and species were singularly tested for hunted wild birds, and appeared to both be significant (*p* < 0.05). As for captured wild birds, the available factors (i.e., sampling area, age, gender, species, individual animal, and order of capture) resulted not significant (*p* > 0.05). Two final models were then constructed to investigate the likelihood of finding an HPAI virus in wild birds based on swab type. Specifically, the hunted wild bird data were modelled considering the two crossed random effect (i.e., the species and the sampling area, to account for the variance among both different bird species and wetlands). One nested random effect (i.e., the order of capture nested in each sampled subject, to account for the variance among different sampled birds and, for each bird, among different catches) was included for captured birds, although both variables were not individually significant in the preliminary analyses to account for the potential re-captures and, thus, for repeated measurements on the same subjects. All the analyses and graphics were performed in R Statistical Software version 4.0.5 [17], using the lme4 [18], emmeans [19] and ggplot2 [20] packages.

*Genome sequencing and phylogenetic analysis*: We sequenced the complete genome of 18 HPAI H5 viruses collected between November 2020 and February 2021, from wild birds and poultry in several Italian regions such as Veneto, Emilia Romagna, Friuli Venezia Giulia and Puglia [https://www.izsvenezie.com/reference–laboratories/avian–influenza–newcastle–disease/italy–update/ accessed on 24 August 2021].

Detailed genetic information for each virus are provided in Supplementary Materials Table S1.

Total RNA was extracted using QIAamp Viral RNA mini kit (QIAGEN, Hilden, Germany); SuperScript™ III One-Step RT-PCR System with Platinum™ Taq High Fidelity DNA Polymerase (Invitrogen, Waltham, MA, USA) was used to amplify whole genomes. Amplicons were purified using Agencourt AMPure XP (Beckman Coulter™, Brea, CA, USA), quantified with Qubit™ DNA HS Assay (Thermo Fisher Scientific, Waltham, MA, USA), and mixed in equimolar proportion. Sequencing libraries were prepared using Illumina Nextera XT DNA Sample Preparation kit; Illumina and sequencing was performed using Illumina MiSeq (2 × 250 bp PE). The read quality was assessed by using FastQC v0.11.2. Complete genomes were generated through a reference-based approach. The reference virus used to obtain the consensus sequences was A/barnacle goose/Germany-SH/AI02167/2020 (A/H5N8) (GISAID accession number: EPI_ISL_614400).

High quality reads were aligned against a reference genome using BWA v0.7.12 [21], processing the alignments with Picard-tools v2.1.0 (http://broadinstitute.github.io/picard/; accessed on 19 October 2021) and GATK v3.5 [21,22,23]. Single nucleotide polymorphisms were called using LoFreq v2.1.2 [24]. Viral sequences were submitted to GISAID EpiFlu Database (Supplementary Material Table S1). Sequences were aligned using MAFFT version 7 [25] (https://mafft.cbrc.jp/alignment/server/; accessed on 24 August 2021). Maximum likelihood phylogenetic tree of the HA gene segment was generated in IQTREE version 1.6, performing ultrafast bootstrap resampling analysis (1000 replications) [26,27]. The phylogenetic tree was visualized by using FigTree version 1.4.2 [http://tree.bio.ed.ac.uk/software/figtree/; accessed on 24 August 2021].

## 3. Results

### 3.1. Surveillance Activity 1 (Hunted Ducks)

The selected wetlands of the activity were sampled at different times from 9 November 2020 to 30 January 2021 (Table 1).

A total of 823 wild waterfowl belonging to 5 different species sampled during the hunting activities, with Eurasian wigeons being the most numerous species sampled (337/823, 40.9%), and Northern pintails the least represented (12/823, 1.5%). All the molecular results for AIV detection obtained within this activity, AIV infection prevalence (%) for each sampling date, hunting valley, avian species and sample material are reported in Table 1. Out of 823 hunted birds, 55 (6.7%) tested overall positive for AIV in at least one out of the three collected samples (OS, CS and FS). Their detailed results for HA-typing, pathotyping, NA-typing are reported in Table 3.

HPAI H5 was first detected in a cloacal swab of a Eurasian wigeon in the Province of Rovigo on 9 November 2020 (Table 1: VCPa-1st); contextually, a LPAI H5Nx virus was observed in a cloacal swab collected from a Eurasian teal.

The highest number of dabbling ducks was sampled between November and December 2020 mainly in VD, VSL, and VCPa wetlands (see DD N. in Table 1), when a concomitant high HPAIV prevalence was observed.

**Figure 2:**
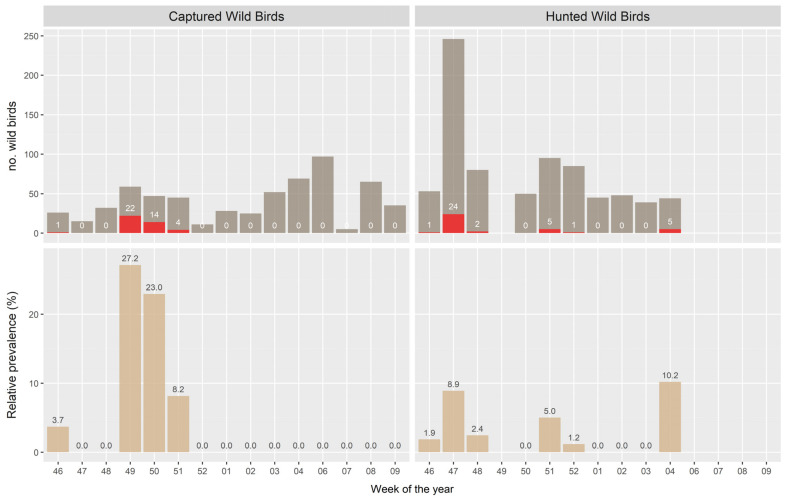
HPAI epidemic curves in captured and hunted wild birds during late autumn–winter seasons 2020–2021 in northeast Italy. Upper panel: number of wild birds tested with RT-PCR; dark grey bars: HPAIV negative birds; red bars: HPAIV positive birds. Lower panel: HPAI relative prevalence.

Apparently, the relative prevalence (Figure 2) in the hunted birds did not follow any noticeable trend, with high prevalence in late November (week 47 in 2020, *p* = 8.9%), mid-December (week 51, 2020, *p* = 5.0%), and finally reaching the highest relative prevalence at the end of the hunting season in late January (week 4, 2021, *p* = 10.2%). However, the type of sampling is not suitable to accurately study the prevalence of AIV infection in the population of interest, and our estimates should not be intended to be reliably used for making inferences on the real magnitude of AIV presence in wild waterfowl.

As shown in Table 2, out of the 100 EWs sampled during the first session in Valle Drago (20-11-21) 10% of OS, 9% of CS and 5% of FS tested positive for H5 HPAI; in the first sampling in Valle San Leonardo (20-11-21), of 54 hunted EWs, the prevalence of HPAI H5 in OS, CS, and FS was 3.7%, 5.6%, and 1.9% respectively; and HPAI H5N8 copresence in OS and CS were found in two DD (Table 3: VLS-1st, H31_EW and H53_EW). Finally, in the second sampling session in VCPa (20-12-15), in the whole group of 89 hunted EWs the HPAI H5 prevalence in OS, CS, and FS was 3.4%, 4.5%, and 1.1%, respectively.

From the second half of December 2020, sporadic detections of HPAIV were observed, and in detail in one OS collected from a Eurasian teal in Valle Morosina at the end of 2020 (Table 3: VM-1st, H47_ET), in two OS from two different duck species (Table 3: VCZ-2nd, H53_NS and H54_ET) and in one FS from an Eurasian wigeon (Table 3: VCZ-2nd, H55_EW) in Valle Ca Zuliani in late January. The copresence of HPAI viruses in OS and FS was also found in one mallard and one Eurasian wigeon (Table 3: VCZ-2nd, H51_Ma and H52_EW) in Valle Ca Zuliani at the end of January.

Eurasian wigeon was the avian species with the highest prevalence of HPAI H5; the majority of individuals that manifested a simultaneous infection of the respiratory and digestive tract (OS + CS) also belong to this species.

The assembled data confirming the positivity of AIVs in the different species of dabbling ducks during the whole surveillance period for activity 1 highlighted the following prevalence in the different biological matrix analyzed (OS, CS, and FS): 2.3%, 1.4%, 0.9% in Eurasian teal, 0%, 0%, 2% in Gadwall, 0%, 0%,0% in Northern pintail, 5%, 5.9%, 3.3% in Eurasian wigeon, 1.8%, 0.9%, 3.6% in Mallard, and finally 1.1%, 3.2%, 1.1% in Northern shoveler.

The high detection of AIVs in swabs taken from the external body surface of sampled ducks is worth mentioning, and can be considered an environmental indicator of the presence of AIVs in the water, also taking into account that ducks already carrying AIV on the body surface could have arrived in our study area [6,28].

In detail, 2 AIVs (no H5, H7, H9 subtypes) and 17 H5 AIVs were detected on the feathers. Although it was not possible to obtain the viral pathotype from five FS, 12 samples were pathotyped as HPAIVs, and five of them were further classified as HPAI H5N8 (Table 3).

Furthermore, one out three selected rRT-PCR AI positive rubbed feather swabs permitted to easily isolate the virus in SPF embryonated chicken eggs (Table 3: H8_Ma, VCZ-1st), confirming the possibility for wild ducks to transport live AIV on the plumage. The oropharyngeal and cloacal swabs submitted for virus isolation in SPF embryonated eggs were also successfully propagated and typed. Selected samples and isolates were analyzed for NGS.

Notably, three different HPAI H5 subtypes were detected. Specifically, the HPAI H5N8 was detected in EW, Ma, and ET in the provinces of Rovigo and Venice, the HPAI H5N5 in an ET in the province of Rovigo and the HPAI H5N1 in EWs in the province of Venice and Rovigo. LPAI viruses were detected in the provinces of Rovigo and/or Venice in ET, EW, Ma, Ga and NS (Table 3).

### 3.2. Survaillance Activity 2 (Captured Ducks)

From November 2020 to March 2021, a total number of 521 dabbling ducks including 374 ET, 79 NP, 61 Ma, 1 CSh, 4 EW, and 2 Ga were sampled and investigated for AIV. All molecular results for AIV detected in captured live birds, AIV prevalence (%) for each trapping/sampling site (VF or VC), and bird species and sample matrix (OS, CS and FS) are reported in Table 2.

A total of 53 aquatic birds tested positive for AIV in at least one out of the three collected samples (OS, CS and FS) (Table 2). It should be noted that all the captured birds were in apparently normal physical and behavioral conditions.

The first positive sample for HPAI H5 was an oropharyngeal swab of a juvenile male Eurasian teal collected on 12 November 2020 (Table 4: C1_ET, VF-1). In the following two sampling sessions only LPAIVs were identified, including a LPAI H5Nx virus from a cloacal swab of an adult female ET (Table 4: C2_ET, VF-2), and AIVs (not belonging to H5, H7, or H9 subtypes) from cloacal swabs of two juvenile male ET (Table 4: C3_ET and C4_ET, VF-3).

An increased number of trapped dabbling ducks was recorded on 4 December 2020 (VF-4) (see DD N. in Table 2), in the overwhelming majority ET, which is the predominant migratory species settling in Valle Figheri. On the same date, we observed the co-circulation of H5N8 and H5N1 HPAI viruses. In total, 22 out of 68 ETs tested positive for HPAI H5; overall 14 ETs tested positive in the upper respiratory tract (OS), 3 ETs were positive in the cloaca (CS), and 9 ETs were positive from the external body surface (FS) (Table 4). Moreover, six Eurasian teals simultaneously showed HPAI H5 in OS and CS (N. 3 ETs), and in OS and FS (N. 3 ETs).

On 11 December 2020 (VF-5), HPAI H5 viruses were mainly found in OS of juvenile female ETs, in one bird a coinfection of HPAI H5N1 and H5N8 was detected (Table 4: C30_ET). HPAIV were identified in VF until 18 December 2020 (VF-6).

Starting from January 2021, only LPAIVs were detected in Valle Figheri and mainly from cloacal swabs of ETs, in particular the detection of a LPAI H7N3 in a cloacal swab of a juvenile female ET (Table 4: C38_ET) and a H9Nx from a feather swab of a juvenile female mallard (Table 4: C39_Ma).

Samples collected in Valle Cavallino (VCa) in February 2021 demonstrated the presence of a LPAI H5Nx in CS of a Common Shelduck, whereas LPAIVs HxNx (negative for H5, H7, and H9) were detected in CS of four Northern Pintail. No HPAIV was found in the wild waterfowl population present at this capture station.

The temporal trend in the relative prevalence at each capture point (Figure 2) was at its highest in the first week of December 2020 (week 49), followed by a decrease in the subsequent two weeks. After the last week of December 2020, no HPAI positive samples were detected in capture birds, despite the increasing number of trapped waterfowl.

**Figure 3:**
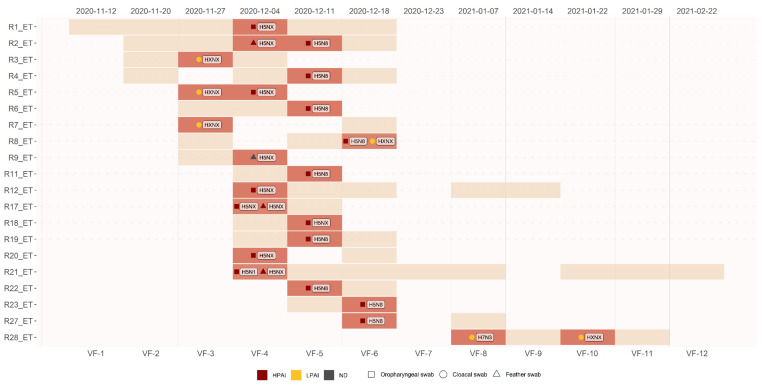
Progression of Avian Influenza virus infections (LPAI and HPAI) in re-captured wild migratory Eurasian teals. Dotted lines: no capture occurred; pale brown bars: bird captured and virologically negative; light red bars: bird captured and virologically positive; dark red symbols: HPAIV; yellow symbols: LPAIV; grey symbols: undetermined pathotype.

During the 14 sampling sessions in VF, several dabbling ducks, identifiable by ring number, were captured at least twice and among them 16 ETs tested HPAIV positive at least once. This allowed us to follow the progression of the HPAI H5 clade 2.3.4.4b infection in naturally infected wild migratory waterfowl (Figure 3).

For 11 recaptured dabbling ducks, it was possible to establish that HPAIV infection occurred during their permanence in this wetland (Figure 3). Most recaptured birds tested positive in either OS or CS with HPAI (H5Nx, H5N8 and/or H5N1), testing both virologically and serologically negative when recaptured approximately one week later (data not shown). Some positive birds R12_ET and R21_ET were recaptured several times, up to 6–9 weeks after the first HPAI H5 detection, and always remaining clinically healthy. A single Eurasian Teal (R8_ET) was simultaneously infected with HPAI H5N8 in OS and an LPAIV (not H5, H7 or H9) in CS. Another Eurasian Teal (R28_ET) was captured four consecutive times, tested positive for LPAI H7N3 in CS (VF-8), then negative (VF-9), positive for AIV in CS (VF-10), and lastly negative for VF-11 (Figure 3). 

Virus isolation was successful from 12 out of 49 samples; in particular 7 influenza viruses from OS, 3 from CS, and 2 from FS were isolated.

### 3.3. Probability of HPAIV Detection

The association between the swab type sampling procedure and the capacity of detecting HPAI viruses in wild bird was explored through two binomial GLMMs. Significant effects were observed only for the model fitted on the captured birds’ data; no significant results were obtained from data on hunted birds. In particular, during surveillance on captured birds, both oropharyngeal and feather swabs showed a positive coefficient significantly higher than the cloacal swabs. This indicated a much higher probability of delivering positive results (i.e., detecting HPAIV) when testing OS or FS, than when testing CS. The contrast of estimates was calculated as the difference between pairs of natural logarithmic coefficient estimates, defined as the ‘log odds ratio’. The assessment of contrasts indicates a significant difference in the performance of OS in comparison with both CS (contrast = 6.79 log odds ratio scale, *p* < 0.0001) and FS (contrast = 4.81 log odds ratio scale, *p* < 0.0001). FS also had a better performance than CS (contrast = 1.99 log odds ratio scale, *p* = 0.031). This allows us to assume that, in the case of HPAI-infected wild dabbling ducks, it is more likely for laboratory tests to deliver positive results from OS or FS rather than from CS.

### 3.4. Genetic Analysis

We analyzed the complete or partial genome of two HPAI H5N1, one HPAI H5N5 and sixteen HPAI H5N8 viruses identified in Italy between November 2020 and February 2021 (analyses are based on sequences produced by the EURL and sequences deposited in GISAID, available on 12 May 2021), (Supplementary Material Table S1). The phylogenetic analysis of the HA gene revealed that all the HPAI H5 viruses analyzed in this study belong to clade 2.3.4.4b and cluster with the HPAI H5 viruses which have been circulating in Europe since October 2020 [2]. No specific mutation associated with mammalian adaptation has been observed in all the sequences analyzed.

The analysis of the eight gene segments shows that the H5N1 viruses cluster with the HPAI H5N1 viruses identified in the Netherlands and Scotland between October 2020 and February 2021; the H5N5 virus groups with HPAI H5N5 viruses detected in Belgium, Germany, Slovenia, Sweden and Wales between October 2020 and March 2021; the H5N8 viruses cluster with the HPAI H5N8 viruses from Europe, Russia, and Kazakhstan (Supplementary Material Figure S1). Interestingly, the Italian viral sequences are interspersed in the phylogenetic trees, suggesting the occurrence of several different introductions likely from northern Europe and Russia. Of note, different introductions of HPAI H5N8 have been observed within the same wetland, more specifically in Valle Drago (Supplementary Material Figure S2).

Furthermore, the HPAI H5N1 and H5N8 viruses detected through passive surveillance for AI in wild birds in Italy (a greater white-fronted goose (*Anser albifrons*), a black-headed gull (*Chroicocephalus ridibundus*) and a greylag goose (*Anser anser*) were found moribund or dead at the end of 2020, and those detected in backyard poultry at the beginning of 2021 were strictly related to the viruses identified in wild birds of these activities. (Supplemental Material Figure S1: A/greater white-fronted goose/Italy/20VIR8073-4/2020 H5N1, A/seagull/Italy/21VIR2479/2021H5N8, A/greylag goose/Italy/20VIR7660-6/2020/H5N8, A/chicken/Italy/21VIR1293-9/2021 H5N8 and A/chicken/Italy/21VIR1151-2/2021 H5N8).

### 3.5. Serological Analyses Results

Seroprevalences by type A influenza virus ELISA were 70.3% (97/138) in Eurasian teal and 77.8% (14/18) in Eurasian wigeon. The reactivity against two HPAI H5 viruses belonging to clade 2.3.4.4b, tested through the hemagglutination inhibition (HI) test, is shown in Table 5. All HI-positive Eurasian teals displayed seroconversion for both antigens, whereas between Eurasian wigeons two birds out of three tested positive for both antigens.

## 4. Discussion

In recent years, we have been witnessing progressive changes in the millennial balance between influenza viruses and their natural reservoir hosts, represented by aquatic birds, Anseriformes in particular. The natural coexistence between these species and LPAIV has been upset by the increasing circulation of HPAI viruses in migratory wild bird populations in the last 15 years, to the point that many species are still paying a very high price for this, such as wild geese and swans, where high HPAI mortality has been recorded in the ongoing epidemic of 2020–2021 [1,2]. Others, such as dabbing ducks, showed greater resistance against H5 HPAI clades that have recently been circulating in Europe and seem to be able to cohabit with these highly pathogenic new variants of AIV as well. The presence of many asymptomatic individuals belonging to these species carrying viruses highly dangerous to other wild and domestic avian species and/or for other susceptible hosts as well, such as mammals including humans, makes the epidemiological scenario of HPAI very worrying and requires the identification of adequate surveillance, and preventive and control strategies.

The results obtained in this paper are particular and reflect a different epidemiological situation to the one observed last winter in northern European countries [1], where a very high number of outbreaks and high mortality in wild birds were observed, and different still from what has been seen in southern Europe, where only very few cases of HPAI in wild birds have been registered [2].

Identifying the introduction and circulation of HPAI viruses in one’s own country, and learning the real extent of the avifauna involvement, is essential for a correct risk analysis and for implementing an adequate package of preventive measures for each country.

Notably, if active surveillance activities had not been conducted on wild birds in Italy, it would not have been possible to measure the real risk to the poultry sector. Other authors [29] had already realized that the role of wild birds in Italy in the introduction of HPAIV in poultry was significantly greater than what it seemed, when considering either the number of wild bird cases or the phylogenetic analyses alone. This showed a poor ability to identify the true level of HPAI infection in wild birds through passive surveillance alone.

The detection of a diffused presence of HPAIV in migratory wintering wild birds already at the beginning of the autumn–winter season led the Italian Ministry of Health to immediately issue a series of risk mitigation measures in high risk areas, such as the ban on outdoor poultry farming, the ban on the use of live decoys for hunting purposes, the intensification of passive and active surveillance (Health Ministerial note n. 0021229-02/10/2020 DGSAF-MDS-P).

Thanks also to these measures, in Italy only three outbreaks in domestic species, all in very small backyard flocks, were registered; this shows the efficacy of such actions if we think that most of the HPAI infected wild birds were found in the wetlands that are mostly located in the proximity of densely populated poultry areas (DPPA).

The arrival of the highest number of infected birds was observed between November and December 2020. At beginning of February 2021, HPAIV were no longer detected, meaning that the period at higher risk of introduction of HPAIV from migratory birds in southern European countries might have been late autumn to early winter.

Introductions of HPAI H5 viruses in a free territory should be closely monitored through existing passive surveillance complemented by active surveillance in selected strategic areas. The migratory behavior of the most important target species should also be taken into account and regarded as an early warning system. The European Commission has listed general criteria for risk-based surveillance in poultry related to virus introductions; however, each country has its own characteristics and peculiarities and require region-specific surveillance and mitigation strategies.

Based on the available data referring to migratory duck species wintering in our sampling areas, it seems that the Eurasian wigeons reported in Italy mostly come from the coasts of northwestern Europe, the United Kingdom, the northern Baltic sites and from large areas along the coasts of the Black and the Caspian Seas, from mainland Russia, Kazakhstan, and as far as the east of the Urals [5].

Most of the overseas recapturing of ringed Eurasian teals in Italy falls within a large geographical area located in northeastern and central European, Baltic, and Scandinavian areas [5,30]. Recaptures of Eurasian teals wintering in Italy were also recorded in eastern Russia up to western Siberia [30]. Such data are in line with what is highlighted by the phylogenetic analyses of the viruses identified in wild ducks during our surveillance activities, from which we have assumed that there have been multiple introductions of HPAIV in the sampling areas, carried by wild birds of different provenience such as northern Europe, Russia, and Kazakhstan.

Our findings confirm previous scientific evidence of Eurasian teal and Eurasian wigeon as long-distance vectors of HPAI H5Nx GsGd viruses 2.3.4.4b, since infection may often occur asymptomatically despite evident oropharyngeal and cloacal viral shedding [31]. Studies conducted in the Netherlands, Korea, and Russia have provided evidence-based proof that these two species acted as long-range vectors of HPAI H5Nx viruses [32,33,34].

Evidence of the simultaneous presence of HPAIV and LPAIV in the same population indicates the high likelihood with which genetic reassortment phenomena may occur in these species, giving rise to new HPAI viral strains [1].

In light of what has been found, it is still very difficult to explain why mortality events were not observed in the HPAI-positive animals or in other species cohabiting or interacting with them (e.g., other waterfowl species, birds of prey, scavengers, etc.). This is even more surprising if we think of the high prevalence of HPAI infection found, with an active replication in the respiratory and digestive tract and a relevant level of contamination on the preened body surface; signs that likely indicate an important environmental presence of the virus in the water [6].

In any case, our results confirm what has been observed in dabbling ducks (Eurasian wigeon, Eurasian teal, and Mallard) and in diving ducks (Common pochard) experimentally infected with HPAI H5N8 2.3.4.4a [31]. The lack of evidence of a specific antibody response against HPAI H5 clade 2.3.4.4b in the vast majority of captured birds serologically tested, is suggestive of the absence of a specific protective herd immunity. In fact, during our surveillance activity only a few birds tested positive for HPAI H5 clade 2.3.4.4b with relatively low HI titers, confirming the results reported by other authors [35].

Despite this, the results obtained in captured and recaptured birds indicate recovery of the infected ducks (Eurasian teals) from HPAIV infection within a few weeks, in the absence of signs of illness and mortality events. Taking into account that experimentally infected ducks produce a scarcely detectable and only transitory antibody response following first exposure to AIV [36], we believe that further studies are needed to better understand if and how the immune response of the reservoir host, including the heterosubtypic one [37], could modulate HPAIV infections.

## 5. Conclusions

Our results highlight that, in order to more efficiently detect the early introduction of novel HPAI viruses, passive surveillance should be complemented by a targeted active surveillance tailored to each country.

In order to optimize resources for the HPAI surveillance in wild birds, careful selection of the areas to be monitored, the species to be sampled, and the samples to be collected is necessary. In particular, our data show that it is more probable to detect H5 HPAI clade 2.3.4.4b in oropharyngeal and feather swabs of dabbling ducks than in cloacal swabs.

Understanding the ecology and evolution of avian influenza in wild birds is crucial in the global perspective of the One Health approach, which should use scientific evidence to implement activities necessary to ensure animal and human health and socioeconomic development, while at the same time minimizing the risks associated with the onset and circulation of new emerging strains.

## Figures and Tables

**Table 1:** Molecular detection and prevalence of AIV in different biological samples collected from hunted dabbling ducks (Veneto region, northeast Italy, 2020–2021). Blue, HPAIV circulation; yellow, LPAIV circulation; green, HPAIV and LPAIV co-circulation.

HVSS ID	Samplingyy-mm-dd	Eurasian Teal				Gadwall				Northern Pintail				Eurasian Wigeon				Mallard				Northern Shoveler				Dabbling Ducks			
		ET	Pos. N. (%)			Ga	Pos. N. (%)			NP	Pos. N. (%)			EW	Pos. N. (%)			Ma	Pos. N. (%)			NS	Pos. N. (%)			DD	Pos. N. (%)		
			OS	CS	FS		OS	CS	FS		OS	CS	FS		OS	CS	FS		OS	CS	FS		OS	CS	FS		OS	CS	FS
VCPa-1st	20-11-09	7	0	1 (14.3)	0	19	0	0	0	1	0	0	0	6	0	2 (33.3)	0	8	0	0	0	13	0	0	0	54	0	3 (5.6)	0
VCZ-1st	20-11-16	1	0	0	0	5	0	0	1 (20)	4	0	0	0	10	1 (10)	2 (20)	0	19	1 (5.3)	1 (5.3)	2 (10.5)	1	0	0	0	40	2 (5)	3 (7.5)	3 (7.5)
VD-1st	20-11-21	0	—	—	—	4	0	0	0	5	0	0	0	100	10 (10)	9 (9)	5 (5)	0	—	—	—	11	0	1 (9.1)	1 (9.1)	120	10 (8.3)	10 (8.3)	6 (5)
VSL-1st	20-11-21	8	0	0	0	0	—	—	—	0	—	—	—	54	2 (3.7)	3 (5.6)	1 (1.9)	8	0	0	0	40	0	0	0	110	2 (1.8)	3 (2.7)	1 (0.9)
VC-1st	20-11-26	32	2 (6.3)	0	2 (6.3)	2	0	0	0	0	—	—	—	16	0	0	2 (12.5)	8	0	0	1 (12.5)	4	0	0	0	62	2 (3.2)	0	5 (8.1)
VD-2nd	20-11-28	0	—	—	—	0	—	—	—	0	—	—	—	20	0	0	—	0	—	—	—	0	—	—	—	20	0	0	—
VCPi	20-12-07	46	1 (2.2)	0	0	0	—	—	—	0	—	—	—	2	0	0	0	2	0	0	0	0	—	—	—	50	1 (2)	0	0
VCPa-2nd	20-12-15	1	0	0	0	3	0	0	0	0	—	—	—	89	3 (3.4)	4 (4.5)	1 (1.1)	5	0	0	0	2	0	0	0	100	3 (3)	4 (4)	1 (1)
VM-1st	20-12-22	60	1 (1.7)	0	0	2	0	0	0	0	—	—	—	0	—	—	—	23	0	0	0	1	0	0	0	86	1 (1.2)	0	0
VC-2nd	21-01-09	20	0	0	0	1	0	0	0	0	—	—	—	16	0	0	0	4	0	0	0	4	0	0	0	45	0	0	0
VD-3rd	21-01-11	15	0	1 (6.7)	0	0	—	—	—	0	—	—	—	15	0	0	0	0	—	—	—	0	—	—	—	30	0	1 (3.3)	0
VSL-2nd	21-01-16	1	0	0	0	0	—	—	—	0	—	—	—	4	0	0	0	6	0	0	0	7	0	1 (14.3)	0	18	0	1 (5.6)	0
VF	21-01-24	1	0	0	0	11	0	0	0	2	0	0	0	2	0	0	0	15	0	0	0	8	0	1 (12.5)	0	39	0	1 (2.6)	0
VCZ-2nd	21-01-25	3	1 (33.3)	0	0	0	—	—	—	0	—	—	—	3	1 (33.3)	0	2 (66.7)	5	1 (20)	0	1 (20)	2	1 (50)	0	0	13	4 (30.8)	0	3 (23.1)
VM-2nd	21-01-30	25	0	1 (4)	0	2	0	0	0	0	—	—	—	0	—	—	—	8	0	0	0	1	0	0	0	36	0	1 (2.8)	0
Total duck & Pos. (%)		220	5 (2.3)	3 (1.4)	2 (0.9)	49	0	0	1 (2)	12	0	0	0	337	17 (5)	20 (5.9)	11 (3.3)	111	2 (1.8)	1 (0.9)	4 (3.6)	94	1 (1.1)	3 (3.2)	1 (1.1)	823	25 (3)	27 (3.3)	19 (2.3)

HPAIV, highly pathogenic avian influenza virus; LPAIV, low pathogenic avian influenza virus; HVSS, hunting valley sampling site; ID, identification; N., Number; ET, Eurasian Teal number; Ga, Gadwall number; NP, Northern Pintail number; EW, Eurasian Wigeon number; Ma, Mallard number; NS, Northern Shoveler number; DD N., Dabbling ducks number; Pos., swabs tested positive by real time RT-PCR for AIV M gene; (%), AIV detection prevalence; OS, oropharyngeal swabs; CS, cloacal swabs; FS, feather swabs; —, sample not available; V., Valle; VCPa, V. Ca Pasta (RO); VCZ, V. Ca Zuliani (RO); VD, V. Drago (VE); VSL, V. San Leonardo (RO); VC, V. Chiusa (RO); VCPi, V. Ca Pisani (RO); VM, V. Morosina (RO); VF, Valle Figheri (VE). Only pathotyped strains have been reported in this table.

**Table 2:** Molecular detection and prevalence of AIV in different biological samples collected from captured dabbling ducks (Veneto region, northItaly, 2020–2021). Blue, HPAIV circulation; yellow, LPAIV circulation; green, HPAIV and LPAIV co-circulation.

TSS ID	Samplingyy-mm-dd	Eurasian Teal				Gadwall				Northern Pintail				Eurasian Wigeon				Mallard				Common Shelduck				Dabbling Ducks			
		ET	Pos. N. (%)			Ga	Pos. N. (%)			NP	Pos. N. (%)			EW	Pos. N. (%)			Ma	Pos. N. (%)			CSh	Pos. N. (%)			DD	Pos. N. (%)		
			OS	CS	FS		OS	CS	FS		OS	CS	FS		OS	CS	FS		OS	CS	FS		OS	CS	FS		OS	CS	FS
VF-1	20-11-12	24	1 (4.2)	0	0	0	—	—	—	0	—	—	—	0	—	—	—	3	0	0	0	0	—	—	—	27	1 (3.7)	0	0
VF-2	20-11-12	10	0	1 (10)	0	0	—	—	—	0	—	—	—	0	—	—	—	3	0	0	0	0	—	—	—	13	0	1 (7.7)	0
VF-3	20-11-27	24	0	2 (8.3)	0	0	—	—	—	0	—	—	—	0	—	—	—	2	0	0	0	0	—	—	—	26	0	2 (7.7)	0
VF-4	20-12-04	68	15 (22.1)	4 (5.9)	9 (13.2)	0	—	—	—	0	—	—	—	0	—	—	—	1	0	0	0	0	—	—	—	69	15 (21.7)	4 (5.8)	9 (13)
VF-5	20-12-11	36	7 (19.4)	0	1 (2.8)	0	—	—	—	0	—	—	—	0	—	—	—	2	0	0	0	0	—	—	—	38	7 (18.4)	0	1 (2.6)
VF-6	20-12-18	24	2 (8.3)	1 (4.2)	0	0	—	—	—	0	—	—	—	0	—	—	—	2	0	0	0	0	—	—	—	26	2 (7.7)	1 (3.8)	0
VF-7	20-12-23	6	0	0	0	0	—	—	—	0	—	—	—	0	—	—	—	1	0	0	0	0	—	—	—	7	0	0	0
VF-8	21-01-07	10	0	1(10)	0	0	—	—	—	0	—	—	—	0	—	—	—	1 *	0	0	0	0	—	—	—	11	0	1 (9.1)	0
VF-9	21-01-14	6	0	0	0	0	—	—	—	0	—	—	—	0	—	—	—	10	0	0	1 (10)	0	—	—	—	16	0	0	1 (6.3)
VF-10	21-01-22	40	0	2 (5)	0	0	—	—	—	0	—	—	—	0	—	—	—	6 **	0	0	0	0	—	—	—	46	0	2 (4.3)	0
VF-11	21-01-29	51	1 (2)	3 (5.9)	0	2	0	0	0	0	—	—	—	0	—	—	—	9	1 (11.1)	0	0	0	—	—	—	62	2 (3.2)	3 (4.8)	0
VF-12	21-02-22	19	0	0	0	0	—	—	—	0	—	—	—	0	—	—	—	2	0	0	0	0	—	—	—	21	0	0	0
VF-13	21-02-25	29	0	2 (6.9)	0	0	—	—	—	0	—	—	—	0	—	—	—	5	0	0	0	0	—	—	—	34	0	2 (5.9)	0
VF-14	21-03-05	9	0	0	0	0	—	—	—	0	—	—	—	0	—	—	—	14	0	0	0	0	—	—	—	23	0	0	0
VCa-1	21-02-12	18	0	0	0	0	—	—	—	74	0	3 (4.1)	0	4	0	0	0	0	—	—	—	1	0	1 (100)	0	97	0	4 (4.1)	0
VCa-2	21-02-15	0	—	—	—	0	—	—	—	5	0	1 (20)	0	0	—	—	—	0	—	—	—	0	—	—	—	5	0	1 (20)	0
Total duck & Pos. (%)		374	26 (7)	16 (4.3)	10 (2.7)	2	0	0	0	79	0	4 (5.1)	0	4	0	0	0	61	1 (1.6)	0	1 (1.6)	1	0	1 (100)	0	521	27 (5.2)	21 (4)	11 (2.1)

HPAIV, highly pathogenic avian influenza virus; LPAIV, low pathogenic avian influenza virus; TSS trapping/sampling site; ID, identification; ET, Eurasian Teal number; Ga, Gadwall number; NP, Northern Pintail number; EW, Eurasian Wigeon number; Ma, Mallard number; CSh, Common Shelduck number; DD N., Dabbling ducks number; Pos., swabs tested positive by real time RT-PCR for AIV M gene; (%), AIV detection prevalence; OS, oropharyngeal swabs; CS, cloacal swabs; FS, feather swabs; —, sample not available; *, domestic form of mallard; **, 1 mallard duck decoy included; VF, Valle Figheri (VE); VCa, Valle Cavallino (VE).Only pathotyped strains have been reported in this table.

**Table 3:** Molecular pathotyping and subtyping of AIV detected in different biological samples collected from hunted dabbling ducks (Veneto region, northeast Italy, 2020–2021).

	AIV Pos			OS Virological Results			CS Virological Results			FS Virological Results		
HVSS ID (yy-mm-dd)	H-Duck ID	Sex	Age	AIV M Gene	AIV P-T	AIV S-T	AIV M Gene	AIV P-T	AIV S-T	AIV M Gene	AIV P-T	AIV S-T
VCPa-1st (20-11-09)	H1_ET	n.a.	n.a.	-	n.d.	n.d.	Pos.	LPAI	H5Nx	-	n.d.	n.d.
	H2_EW	n.a.	n.a.	-	n.d.	n.d.	**Pos.**	**HPAI**	**H5Nx**	-	n.d.	n.d.
	H3_EW	n.a.	n.a.	-	n.d.	n.d.	Pos.	LPAI	H9Nx	-	n.d.	n.d.
VCZ-1st (20-11-16)	H4_EW	n.a.	n.a.	-	n.d.	n.d.	**Pos.**	**HPAI**	**H5Nx**	-	n.d.	n.d.
	H5_EW	n.a.	n.a.	**Pos.**	**HPAI**	**H5N8**	**Pos.**	**HPAI**	**H5Nx**	-	n.d.	n.d.
	H6_Ga	n.a.	n.a.	-	n.d.	n.d.	-	n.d.	n.d.	Pos.	LPAI	HxNx
	H7_Ma	n.a.	n.a.	-	n.d.	n.d.	-	n.d.	n.d.	Pos.	LPAI	HxNx
	H8_Ma	n.a.	n.a.	-	n.d.	n.d.	-	n.d.	n.d.	**Pos.**	**HPAI**	**H5N8**
	H9_Ma	n.a.	n.a.	**Pos.**	**HPAI**	**H5N8**	**Pos.**	**HPAI**	**H5N8**	-	n.d.	n.d.
VD-1st (20-11-21)	H10_EW	F	J	**Pos.**	**HPAI**	**H5N8**	**Pos.**	**HPAI**	**H5Nx**	-	n.d.	n.d.
	H11_EW	F	J	**Pos.**	**HPAI**	**H5Nx**	**Pos.**	**HPAI**	**H5Nx**	-	n.d.	n.d.
	H12_EW	F	A	Pos.	n.a.	H5Nx	-	n.d.	n.d.	-	n.d.	n.d.
	H13_EW	F	J	-	n.d.	n.d.	-	n.d.	n.d.	**Pos.**	**HPAI**	**H5Nx**
	H14_EW	M	A	-	n.d.	n.d.	Pos.	n.a.	H5Nx	-	n.d.	n.d.
	H15_EW	F	J	**Pos.**	**HPAI**	**H5N8**	-	n.d.	n.d.	-	n.d.	n.d.
	H16_EW	F	J	**Pos.**	**HPAI**	**H5N8**	-	n.d.	n.d.	-	n.d.	n.d.
	H17_EW	F	J	-	n.d.	n.d.	**Pos.**	**HPAI**	**H5Nx**	-	n.d.	n.d.
	H18_EW	M	A	-	n.d.	n.d.	-	n.d.	n.d.	**Pos.**	**HPAI**	**H5Nx**
	H19_EW	F	J	**Pos.**	**HPAI**	**H5Nx**	Pos.	n.a.	H5Nx	-	n.d.	n.d.
	H20_EW	M	J	Pos.	n.a.	H5Nx	-	n.d.	n.d.	-	n.d.	n.d.
	H21_EW	M	J	-	n.d.	n.d.	**Pos.**	**HPAI**	**H5Nx**	-	n.d.	n.d.
	H22_EW	F	J	**Pos.**	**HPAI**	**H5N8**	-	n.d.	n.d.	-	n.d.	n.d.
	H23_EW	M	A	-	n.d.	n.d.	-	n.d.	n.d.	**Pos.**	**HPAI**	**H5Nx**
	H24_EW	M	J	**Pos.**	**HPAI**	**H5Nx**	**Pos.**	**HPAI**	**H5Nx**	**Pos.**	**HPAI**	**H5Nx**
	H25_EW	F	A	**Pos.**	**HPAI**	**H5Nx**	-	n.d.	n.d.	-	n.d.	n.d.
	H26_EW	M	J	-	n.d.	n.d.	Pos.	n.a.	H5Nx	-	n.d.	n.d.
	H27_EW	M	J	-	n.d.	n.d.	**Pos.**	**HPAI**	**H5N1**	-	n.d.	n.d.
	H28_EW	M	J	-	n.d.	n.d.	-	n.d.	n.d.	**Pos.**	**HPAI**	**H5Nx**
	H29_NS	F	J	-	n.d.	n.d.	**Pos.**	**HPAI**	**H5Nx**	-	n.d.	n.d.
	H30_NS	F	J	-	n.d.	n.d.	-	n.d.	n.d.	**Pos.**	**HPAI**	**H5Nx**
VSL-1st (20-11-21)	H31_EW	n.a.	n.a.	**Pos.**	**HPAI**	**H5N8**	**Pos.**	**HPAI**	**H5N8**	-	n.d.	n.d.
	H32_EW	n.a.	n.a.	-	n.d.	n.d.	Pos.	n.a.	H5Nx	**Pos.**	**HPAI**	**H5N8**
	H33_EW	n.a.	n.a.	**Pos.**	**HPAI**	**H5N8**	**Pos.**	**HPAI**	**H5N8**	-	n.d.	n.d.
VC-1st (20-11-26)	H34_Ma	n.a.	n.a.	-	n.d.	n.d.	-	n.d.	n.d.	Pos.	n.a.	H5Nx
	H35_ET	n.a.	n.a.	**Pos.**	**HPAI**	**H5N8**	-	n.d.	n.d.	-	n.d.	n.d.
	H36_ET	n.a.	n.a.	-	n.d.	n.d.	-	n.d.	n.d.	Pos.	n.a.	H5Nx
	H37_ET	n.a.	n.a.	-	n.d.	n.d.	-	n.d.	n.d.	Pos.	n.a.	H5Nx
	H38_ET	n.a.	n.a.	**Pos.**	**HPAI**	**H5N5**	-	n.d.	n.d.	-	n.d.	n.d.
	H39_EW	n.a.	n.a.	-	n.d.	n.d.	-	n.d.	n.d.	Pos.	n.a.	H5Nx
	H40_EW	n.a.	n.a.	-	n.d.	n.d.	-	n.d.	n.d.	Pos.	n.a.	H5Nx
VCPi (20-12-07)	H41_ET	n.a.	n.a.	Pos.	LPAI	HxNx	-	n.d.	n.d.	-	n.d.	n.d.
VCPa-2nd (20-12-15)	H42_EW	n.a.	n.a.	**Pos.**	**HPAI**	**H5N8**	**Pos.**	**HPAI**	**H5N8**	-	n.d.	n.d.
	H43_EW	n.a.	n.a.	**Pos.**	**HPAI**	**H5N8**	**Pos.**	**HPAI**	**H5N8**	**Pos.**	**HPAI**	**H5N8**
	H44_EW	n.a.	n.a.	**Pos.**	**HPAI**	**H5N1**	-	n.d.	n.d.	-	n.d.	n.d.
	H45_EW	n.a.	n.a.	-	n.d.	n.d.	**Pos.**	**HPAI**	**H5N1**	-	n.d.	n.d.
	H46_EW	n.a.	n.a.	-	n.d.	n.d.	**Pos.**	**HPAI**	**H5N1**	-	n.d.	n.d.
VM-1st (20-12-22)	H47_ET	n.a.	n.a.	**Pos.**	**HPAI**	**H5N8**	-	n.d.	n.d.	-	n.d.	n.d.
VD-3rd (21-01-11)	H48_ET	M	J	-	n.d.	n.d.	Pos.	LPAI	HxNx	-	n.d.	n.d.
VSL-2nd (21-01-16)	H49_NS	n.a.	n.a.	-	n.d.	n.d.	Pos.	LPAI	HxNx	-	n.d.	n.d.
VF (21-01-24)	H50_NS	M	J	-	n.d.	n.d.	Pos.	LPAI	HxNx	-	n.d.	n.d.
VCZ-2nd (21-01-25)	H51_Ma	n.a.	n.a.	**Pos.**	**HPAI**	**H5N8**	-	n.d.	n.d.	**Pos.**	**HPAI**	**H5N8**
	H52_EW	n.a.	n.a.	**Pos.**	**HPAI**	**H5N8**	-	n.d.	n.d.	**Pos.**	**HPAI**	**H5N8**
	H53_NS	n.a.	n.a.	**Pos.**	**HPAI**	**H5N8**	-	n.d.	n.d.	-	n.d.	n.d.
	H54_ET	n.a.	n.a.	**Pos.**	**HPAI**	**H5N8**	-	n.d.	n.d.	-	n.d.	n.d.
	H55_EW	n.a.	n.a.	-	n.d.	n.d.	-	n.d.	n.d.	**Pos.**	**HPAI**	**H5N8**
VM-2nd (21-01-30)	H56_ET	n.a.	n.a.	-	n.d.	n.d.	Pos.	LPAI	HxNx	-	n.d.	n.d.

HVSS, hunting valley sampling site; ID, identification; H-duck, hunted-duck; AIV Pos., avian influenza virus swabs tested positive by real time RT-PCR for AIV M gene; OS, oropharyngeal swabs; CS, cloacal swabs; FS, feather swabs; P-T, pathotype; S-T, subtype; n.a., not applicable, n.d.; not done; V, Valle; VCPa, V. Ca Pasta (RO); VCZ, V. Ca Zuliani (RO); VD, V. Drago (VE); VSL, V. San Leonardo (RO); VC, V. Chiusa (RO); VCPi, V. Ca Pisani (RO); VM, V. Morosina (RO); VF, Valle Figheri (VE); ET, Eurasian Teal; EW, Eurasian Wigeon; Ga, Gadwall; Ma, Mallard; NS, Northern Shoveler; LPAI, low pathogenic avian influenza; HPAI, highly pathogenic avian influenza (in bold font).

**Table 4:** Molecular pathotyping and subtyping of AIV detected in different biological samples collected from captured dabbling ducks (Veneto region, northeast Italy, 2020–2021).

	AIV Pos			OS Virological Results			CS Virological Results			FS Virological Results		
TSS ID (yy-mm-dd)	C-Duck ID	Sex	Age	AIV M Gene	AIV P-T	AIV S-T	AIV M Gene	AIV P-T	AIV S-T	AIV M Gene	AIV P-T	AIV S-T
VF-1 (20-11-12)	C1_ET	M	J	**Pos.**	**HPAI**	**H5Nx**	-	n.d.	n.d.	-	n.d.	n.d.
VF-2 (20-11-20)	C2_ET	F	A	-	n.d.	n.d.	Pos.	LPAI	H5Nx	-	n.d.	n.d.
VF-3 (20-11-27)	C3_ET	M	J	-	n.d.	n.d.	Pos.	LPAI	HxNx	-	n.d.	n.d.
	C4_ET	M	J	-	n.d.	n.d.	Pos.	LPAI	HxNx	-	n.d.	n.d.
VF-4 (20-12-04)	C5_ET	F	J	**Pos.**	**HPAI**	**H5Nx**	-	n.d.	n.d.	-	n.d.	n.d.
	C6_ET	M	J	**Pos.**	**HPAI**	**H5Nx**	-	n.d.	n.d.	-	n.d.	n.d.
	C7_ET	F	J	**Pos.**	**HPAI**	**H5Nx**	-	n.d.	n.d.	-	n.d.	n.d.
	C8_ET	F	J	**Pos.**	**HPAI**	**H5Nx**	**Pos.**	**HPAI**	**H5Nx**	-	n.d.	n.d.
	C9_ET	F	J	**Pos.**	**HPAI**	**H5Nx**	-	n.d.	n.d.	**Pos.**	**HPAI**	**H5Nx**
	C10_ET	F	A	Pos.	LPAI	HxNx	-	n.d.	n.d.	-	n.d.	n.d.
	C11_ET	F	A	**Pos.**	**HPAI**	**H5Nx**	-	n.d.	n.d.	-	n.d.	n.d.
	C12_ET	M	J	**Pos.**	**HPAI**	**H5N8**	**Pos.**	**HPAI**	**H5Nx**	-	n.d.	n.d.
	C13_ET	M	A	**Pos.**	**HPAI**	**H5Nx**	-	n.d.	n.d.	-	n.d.	n.d.
	C14_ET	F	J	**Pos.**	**HPAI**	**H5Nx**	-	n.d.	n.d.	-	n.d.	n.d.
	C15_ET	M	J	**Pos.**	**HPAI**	**H5N8**	**Pos.**	**HPAI**	**H5Nx**	-	n.d.	n.d.
	C16_ET	F	J	-	n.d.	n.d.	Pos.	LPAI	HxNx	-	n.d.	n.d.
	C17_ET	F	J	**Pos.**	**HPAI**	**H5Nx**	-	n.d.	n.d.	-	n.d.	n.d.
	C18_ET	F	J	**Pos.**	**HPAI**	**H5Nx**	-	n.d.	n.d.	**Pos.**	**HPAI**	**H5Nx**
	C19_ET	F	J	**Pos.**	**HPAI**	**H5Nx**	-	n.d.	n.d.	-	n.d.	n.d.
	C20_ET	M	A	**Pos.**	**HPAI**	**H5N1**	-	n.d.	n.d.	**Pos.**	**HPAI**	**H5Nx**
	C21_ET	M	J	-	n.d.	n.d.	-	n.d.	n.d.	**Pos.**	**HPAI**	**H5Nx**
	C22_ET	F	J	-	n.d.	n.d.	-	n.d.	n.d.	**Pos.**	**HPAI**	**H5Nx**
	C23_ET	M	J	-	n.d.	n.d.	-	n.d.	n.d.	**Pos.**	**HPAI**	**H5Nx**
	C24_ET	F	A	-	n.d.	n.d.	-	n.d.	n.d.	**Pos.**	**HPAI**	**H5Nx**
	C25_ET	F	J	-	n.d.	n.d.	-	n.d.	n.d.	**Pos.**	**HPAI**	**H5Nx**
	C26_ET	F	J	-	n.d.	n.d.	-	n.d.	n.d.	**Pos.**	**HPAI**	**H5Nx**
VF-5 (20-12-11)	C27_ET	M	J	**Pos.**	**HPAI**	**H5N8**	-	n.d.	n.d.	-	n.d.	n.d.
	C28_ET	F	J	**Pos.**	**HPAI**	**H5N8**	-	n.d.	n.d.	-	n.d.	n.d.
	C29_ET	F	J	**Pos.**	**HPAI**	**H5N8**	-	n.d.	n.d.	-	n.d.	n.d.
	C30_ET	F	J	**Pos.**	**HPAI**	**H5N1-N8**	-	n.d.	n.d.	-	n.d.	n.d.
	C31_ET	F	A	**Pos.**	**HPAI**	**H5N8**	-	n.d.	n.d.	-	n.d.	n.d.
	C32_ET	F	J	**Pos.**	**HPAI**	**H5N8**	-	n.d.	n.d.	-	n.d.	n.d.
	C33_ET	F	J	**Pos.**	n.a.	**H5N8**	-	n.d.	n.d.	-	n.d.	n.d.
	C34_ET	F	J	-	n.d.	n.d.	-	n.d.	n.d.	**Pos.**	**HPAI**	**H5N8**
VF-6 (20-12-18)	C35_ET	F	J	**Pos.**	**HPAI**	**H5N8**	-	n.d.	n.d.	-	n.d.	n.d.
	C36_ET	M	J	**Pos.**	**HPAI**	**H5N8**	-	n.d.	n.d.	-	n.d.	n.d.
	C37_ET	F	J	-	n.d.	n.d.	Pos.	LPAI	HxNx	-	n.d.	n.d.
VF-8 (21-01-07)	C38_ET	F	J	-	n.d.	n.d.	Pos.	LPAI	H7N3	-	n.d.	n.d.
VF-9 (21-01-14)	C39_Ma	F	J	-	n.d.	n.d.	-	n.d.	n.d.	Pos.	LPAI	H9Nx
VF-10 (21-01-22)	C40_ET	F	J	-	n.d.	n.d.	Pos.	LPAI	HxNx	-	n.d.	n.d.
	C41_ET	F	J	-	n.d.	n.d.	Pos.	LPAI	HxNx	-	n.d.	n.d.
VF-11 (21-01-29)	C42_ET	M	J	Pos.	LPAI	HxNx	-	n.d.	n.d.	-	n.d.	n.d.
	C43_ET	F	J	-	n.d.	n.d.	Pos.	LPAI	HxNx	-	n.d.	n.d.
	C44_ET	M	J	-	n.d.	n.d.	Pos.	LPAI	HxNx	-	n.d.	n.d.
	C45_ET	F	J	-	n.d.	n.d.	Pos.	LPAI	HxNx	-	n.d.	n.d.
	C46_Ma	M	J	Pos.	LPAI	HxNx	-	n.d.	n.d.	-	n.d.	n.d.
VF-13 (21-02-25)	C47_ET	F	J	-	n.d.	n.d.	Pos.	LPAI	HxNx	-	n.d.	n.d.
	C48_ET	M	J	-	n.d.	n.d.	Pos.	LPAI	HxNx	-	n.d.	n.d.
VCa-1 (21-02-12)	C49_CSh	F	J	-	n.d.	n.d.	Pos.	LPAI	H5Nx	-	n.d.	n.d.
	C50_NP	A	M	-	n.d.	n.d.	Pos.	LPAI	H5Nx	-	n.d.	n.d.
	C51_NP	A	F	-	n.d.	n.d.	Pos.	LPAI	H5Nx	-	n.d.	n.d.
	C52_NP	J	F	-	n.d.	n.d.	Pos.	LPAI	H5Nx	-	n.d.	n.d.
VCa-1 (21-02-15)	C53_NP	n.a.	n.a.	-	n.d.	n.d.	Pos.	LPAI	HxNx	-	n.d.	n.d.

TSS, trapping/sampling site; ID, identification; C-duck, captured-duck; AIV Pos., avian influenza virus swabs tested positive by real time RT-PCR for AIV M gene; OS, oropharyngeal swabs; CS, cloacal swabs; FS, feather swabs; P-T, pathotype; S-T, subtype; n.a., not applicable, n.d.; not done; VF, Valle Figheri (VE); VCa, V. Cavallino (VE); ET, Eurasian Teal; Ma, Mallard; CSh, Common Shelduck; NP, Northern Pintail; LPAI, low pathogenic avian influenza; HPAI, highly pathogenic avian influenza (in bold font).

**Table 5:** H5 HPAI HI reactivity in type A Influenza ELISA-positive sera.

HPAIV Antigen	HI Titers in ELISA-Positive Ducks	Duck Species (Wetland)	
		Eurasian Teal (VF)	Eurasian Wigeon (VD)
H5N8/2020 (*) Clade 2.3.4.4b	<1:16	93	11
	1:16	0	2
	1:32	2	1
	1:64	2	0
	1:128	0	0
	seroprevalence (%)	4/138 (2.9%)	3/18 (16.7%)
H5N5/2016 (§) Clade 2.3.4.4b	<1:16	93	12
	1:16	2	0
	1:32	1	1
	1:64	1	1
	1:128	0	0
	seroprevalence (%)	4/138 (2.9%)	2/18 (11.1%)

Strains used as HA antigens: (*) H5N8/2020, A/mute swan/Netherlands/01/20 (H5N8); (§) H5N5/2016, A/wigeon/Italy/16VIR9616-3/16 (H5N5); VF, Valle Figheri; VD, Valle Drago. HI titers lower than 1:16 were considered negative.

## Data Availability

Not applicable.

## Supplementary Materials

The following are available online at https://www.mdpi.com/article/10.3390/microorganisms9112188/s1: Table S1: H5 HPAI viruses analyzed for phylogenetic analysis, viral sequences were submitted to GISAID EpiFlu Database; Figure S1: Maximum Likelihood phylogenetic tree of the **HA** gene obtained using IQTREE v1.6.6. Ultrafast bootstrap supports higher than 80 are indicated next to the nodes. The Italian virus are colored according to Avian Influenza subtypes; Figure S2: Maximum Likelihood phylogenetic tree of the **HA** gene obtained using IQTREE v1.6.6. Ultrafast bootstrap supports higher than 80 are indicated next to the nodes. The Italian virus are colored according to the selected wetland.
