# Supplementary material for: Active Surveillance for Highly Pathogenic Avian Influenza Viruses in Wintering Waterbirds in Northeast Italy, 2020–2021
Source: Microorganisms. 2021 Oct 20;9(11):2188. doi: 10.3390/microorganisms9112188 (PMC8621713; doi:10.3390/microorganisms9112188)
Supplement: Supplementary file 1 [file microorganisms-09-02188-s001.zip › microorganisms-1410352-supplementary/Table S1.pdf]

microorganisms MDPI

1
Table S1. H5 HPAI viruses analysed for phylogenetic analysis, viral sequences were submitted to GISAID EpiFlu Database 2

Virus	H5 HPAI Subtype clade 2.3.4.4b	Data collection	Region	Province	Location	Accession Number
**A/Eurasian_wigeon/Italy/20VIR7139-121/2020**	H5N8	2020-11-14	Veneto	Rovigo	Valle Ca' Zuliani	EPI1815147-EPI1815154
**A/mallard/Italy/20VIR7139-124_feather/2020**	H5N8	2020-11-14	Veneto	Rovigo	Valle Ca' Zuliani	EPI1815156-EPI1815163
**A/mallard/Italy/20VIR7139-73/2020**	H5N8	2020-11-14	Veneto	Rovigo	Valle Ca' Zuliani	EPI1814603-EPI1814610
A/common_buzzard/Italy/21VIR431-7/2020	H5N8	2020-11-17	Puglia	Lecce	Lecce	EPI1858349-EPI1858356
**A/Eurasian_wigeon/Italy/20VIR7301-362/2020**	H5N8	2020-11-21	Veneto	Venezia	Valle Drago - Jesolo	EPI1843643-EPI1843650
**A/Eurasian_wigeon/Italy/20VIR7301-206/2020**	H5N1	2020-11-21	Veneto	Venezia	Valle Drago - Jesolo	EPI1815139-EPI1815146
**A/Eurasian_wigeon/Italy/20VIR7301-31/2020**	H5N8	2020-11-21	Veneto	Venezia	Valle Drago - Jesolo	EPI1815378-EPI1815385
**A/Eurasian_wigeon/Italy/20VIR7301-34/2020**	H5N8	2020-11-21	Veneto	Venezia	Valle Drago - Jesolo	EPI1815371-EPI1815377
A/greater_white-fronted_goose/Italy/20VIR8073-4/2020	H5N1	2020-11-23	FVG	Gorizia	Monfalcone	EPI1843635-EPI1843642
**A/common_teal/Italy/20VIR7439-191/2020**	H5N8	2020-11-28	Veneto	Rovigo	Valle Chiusa	EPI1858205-EPI1858212
**A/common_teal/Italy/20VIR7439-190/2020**	H5N5	2020-11-28	Veneto	Rovigo	Valle Chiusa	EPI1843619-EPI1843626

**Tab.S1** *(cont.)*						
A/greylag_goose/Italy/20VIR7660-6/2020	H5N8	2020-11-29	Veneto	Venezia	Jesolo	EPI1843627-EPI1843634
**A/common_teal/Italy/20VIR7608-73/2020**	H5N8	2020-12-04	Veneto	Venezia	Valle Figheri	EPI1843611-EPI1843618
A/ornamental_grey_crowned_crane/Italy/21VIR436/2021	H5N8	2021-01-19	Emilia Romagna	Ravenna	Lugo	na
A/seagull/Italy/21VIR2479/2021	H5N8	2021-01-28	Emilia Romagna	Rimini	Spiaggia Viserba	EPI1860048-EPI1860055
A/chicken/Italy/21VIR1151-2/2021	H5N8	2021-02-17	FVG	Pordenone	Chions	EPI1858173-EPI1858180
A/duck/Italy/21VIR1293-15/2021	H5N8	2021-02-23	Veneto	Padova	San Giorgio delle Pertiche	EPI1858213-EPI1858220
A/guinea_fowl/Italy/21VIR1293-20/2021	H5N8	2021-02-23	Veneto	Padova	San Giorgio delle Pertiche	EPI1858229-EPI1858236
A/chicken/Italy/21VIR1293-9/2021	H5N8	2021-02-23	Veneto	Padova	San Giorgio delle Pertiche	EPI1858181-EPI1858188

bold viral strains isolated in migratory birds during active surveillance activities
