# Supplementary figures and images for: Active Surveillance for Highly Pathogenic Avian Influenza Viruses in Wintering Waterbirds in Northeast Italy, 2020–2021
Source: Microorganisms. 2021 Oct 20;9(11):2188. doi: 10.3390/microorganisms9112188 (PMC8621713; doi:10.3390/microorganisms9112188)

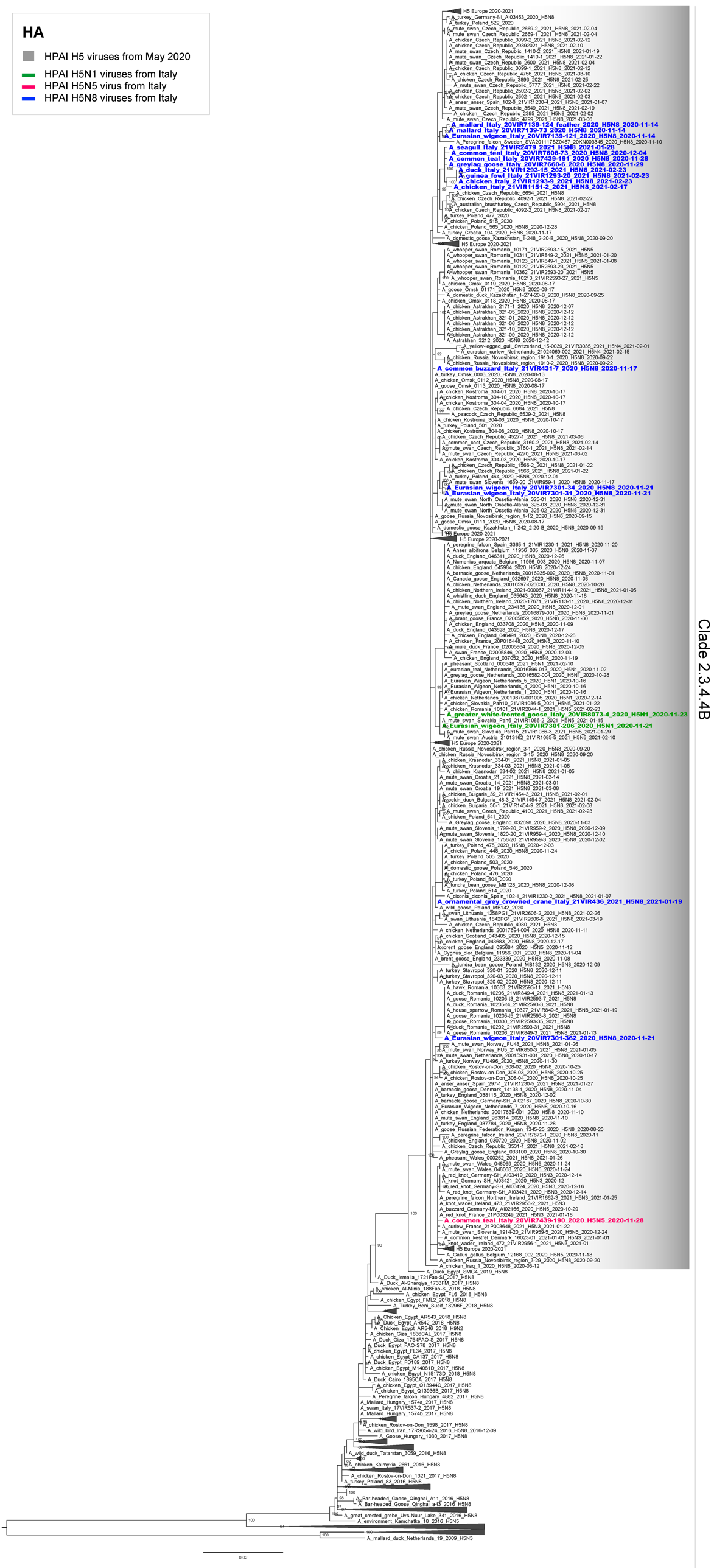

Supplement: Supplementary file 1 [file microorganisms-09-02188-s001.zip › microorganisms-1410352-supplementary/Figure S1.png]

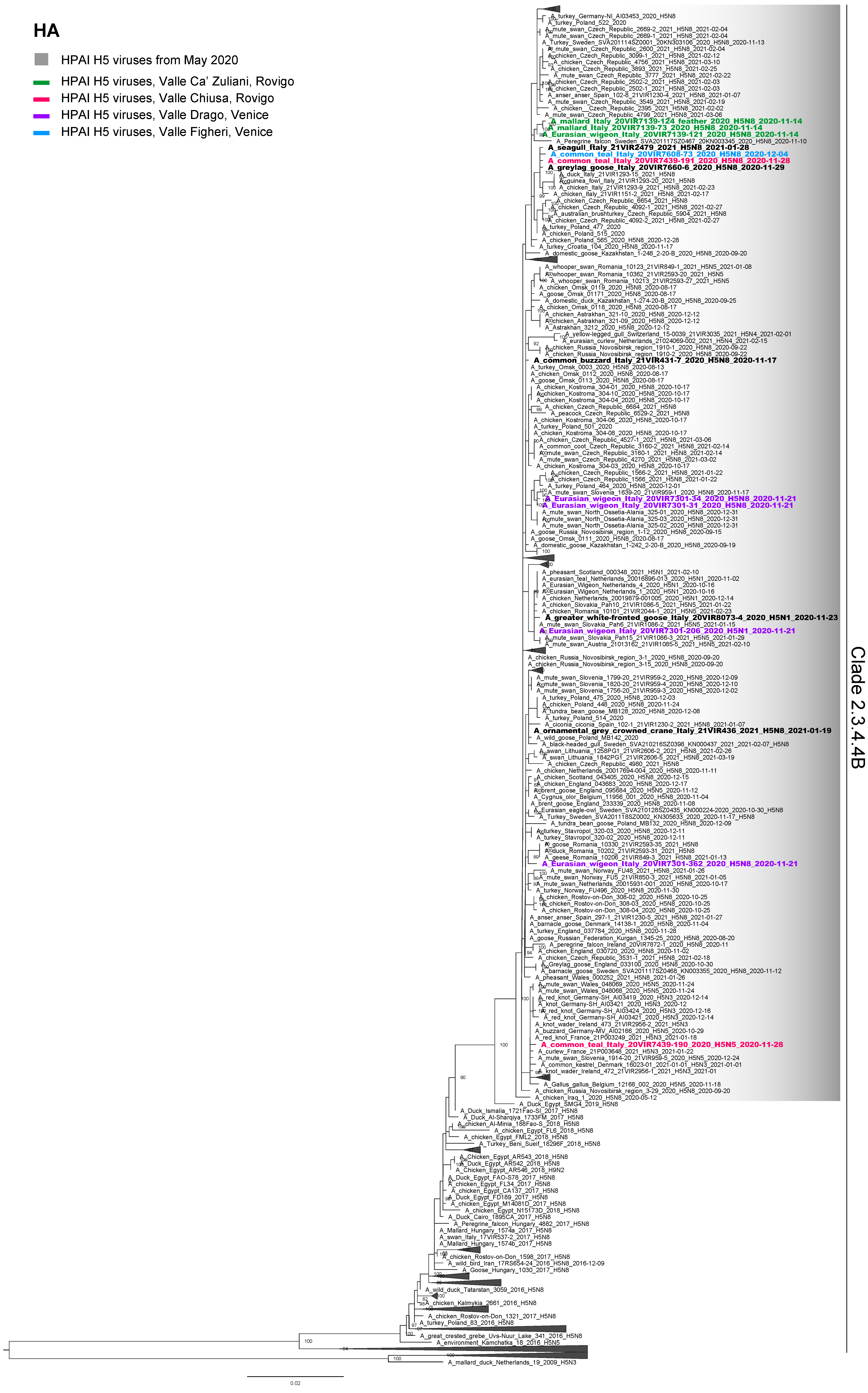

Supplement: Supplementary file 1 [file microorganisms-09-02188-s001.zip › microorganisms-1410352-supplementary/Figure S2.png]
